# Investigation of the cumulative number of chromosome aberrations induced by three consecutive CT examinations in eight patients

**DOI:** 10.1093/jrr/rrz068

**Published:** 2019-10-28

**Authors:** Yu Abe, Hideyoshi Noji, Tomisato Miura, Misaki Sugai, Yumiko Kurosu, Risa Ujiie, Naohiro Tsuyama, Aki Yanagi, Yukari Yanai, Takashi Ohba, Tetsuo Ishikawa, Kenji Kamiya, Mitsuaki A Yoshida, Akia Sakai

**Affiliations:** 1 Department of Radiation Life Sciences, Fukushima Medical University School of Medicine, Fukushima, Japan; 2 Department of Medical Oncology, Fukushima Medical University School of Medicine, Fukushima, Japan; 3 Department of Bioscience and Laboratory Medicine, Hirosaki University Graduate School of Health Sciences, Hirosaki, Japan; 4 Radiation Medical Science Center for the Fukushima Health Management Survey, Fukushima Medical University, Fukushima, Japan; 5 Department of Radiation Health Management, Fukushima Medical University School of Medicine, Fukushima, Japan; 6 Department of Radiation Physics and Chemistry, Fukushima Medical University School of Medicine, Fukushima, Japan; 7 Department of Experimental Oncology, Research Institute for Radiation Biology and Medicine, Hiroshima University, Hiroshima, Japan; 8 Department of Radiation Biology, Institute of Radiation Emergency Medicine, Hirosaki University, Hirosaki, Japan

**Keywords:** chromosome aberration, dicentric chromosome, chromosome translocation, computed tomography, effective radiation dose

## Abstract

In our previous study, we found that chromosomes were damaged by the radiation exposure from a single computed tomography (CT) examination, based on an increased number of dicentric chromosomes (Dics) formed in peripheral blood lymphocytes after a CT examination. We then investigated whether a cumulative increase in the frequency of Dics and chromosome translocations (Trs) formation could be observed during three consecutive CT examinations performed over the course of 3–4 years, using lymphocytes in peripheral bloods of eight patients (five males and three females; age range 27–77 years; mean age, 64 years). The effective radiation dose per CT examination estimated from the computational dosimetry system was 22.0–73.5 mSv, and the average dose per case was 40.5 mSv. The frequency of Dics formation significantly increased after a CT examination and tended to decrease before the next examination. Unlike Dics analysis, we found no significant increase in the frequency of Trs formation before and after the CT examination, and we observed no tendency for the frequency to decrease before the next CT examination. The frequency of Trs formation was higher than that of Dics formation regardless of CT examination. Furthermore, neither analysis of Dics nor Trs showed a cumulative increase in the frequency of formation following three consecutive CT examinations.

## INTRODUCTION

The annual radiation exposure dose of the Japanese population is about 2.1 mSv, which is derived from natural sources, plus about 3.9 mSv, which is derived from artificial ionizing radiation. Almost all of the latter is due to medical radiation exposure, and the majority of it is due to computed tomography (CT) examination [1, 2]. CT examination is a very useful diagnostic method and its use has increased rapidly. However, the radiation exposure dose of abdominal CT examination in an adult is around 20 mSv, and the relationship between radiation exposure from frequent CT examinations and the prevalence of cancer is a concern [3, 4]. Pearce *et al*. reported that CT examination is a risk factor for development of leukemia and brain tumors in children [5]. Furthermore, the prevalence of cancer is higher in a group that underwent CT examination compared with a group that did not, according to a cohort study of 11 million children and adolescents [6]. On the other hand, after excluding children with congenital chromosomal aberrations and immunodeficiency such as HIV infection, which are cancer-predisposing factors, no significant increase in the prevalence of cancer was observed [7]. In previous studies, we analyzed dicentric chromosomes (Dics) formation and chromosome translocations (Trs) to determine whether such Dics and Trs are induced by ionizing radiation exposure due to CT examination [8, 9]. We showed an increase in Dics formation after a single CT scan (5.57–60.27 mSv: mean 24.24 mSv), but the frequency of Trs detected before and after the CT scan did not differ significantly. Therefore, the data suggested that DNA double-strand breaks (DSBs) could be induced by the radiation exposure from one CT scan. However, an increase in Trs was not likely to be detected because of the high baseline due to multiple confounding factors in adults. We here investigated whether cumulative Dics and Trs formation could be observed during three consecutive CT examinations performed over the course of 3–4 years, using lymphocytes in peripheral blood (PB).

## MATERIALS AND METHODS

### Ethics statement

The samples and the medical records used in our study have been approved by the Ethics Committee of the Fukushima Medical University School of Medicine (approval number 1577). Written informed consent was obtained from all participants for analysis of PB samples, and the methods were carried out in accordance with approved guidelines of the Council for International Organizations of Medical Science [10].

### Blood donors

PB samples were collected from eight patients (five male and three female) aged 27–77 years (mean 64 years) who were followed in the Department of Hematological Internal Medicine at Fukushima Medical University Hospital. A total of six samples were collected from the same patient for analyses before and after three consecutive CT examinations. The follow-up was approximately 1 year after each CT examination.

### Lymphocyte culture

Blood samples were taken just before and within a month after each CT examination (Table 1). Mononuclear blood cells were isolated from heparinized PB from each sample using BD Vacutainer CPT tubes (BD Biosciences, San Jose, CA, USA) according to the manufacturer’s instructions. Cells were suspended in RPMI 1640 medium (Nacalai Tesque, Kyoto, Japan) containing 20% fetal bovine serum (Equitech Bio, Keilor East, Australia), 2% phytohaemagglutinin-HA15 (Remel, Lenexa, KS, USA) and 60 μg/ml kanamycin solution (Life Technologies, Carlsbad, CA, USA) in a tube. Lymphocytes were cultured in a 5% humidified CO_2_ incubator at 37°C for 46 h. Then, colcemid solution (Wako, Osaka, Japan) was added (final concentration: 50 ng/ml or 0.05 μg/ml) and cells were cultured for an additional 2 h. After 48 h of culture, chromosome preparations were made according to the standard cytogenetic procedure [11].

**Table 1:** Patient characteristics

Patient No	Sex	Age	Disease	Part of body examined in CT scan	Period between 1^st^ and 2nd (days)	Period between 2nd and 3rd (days)	Days from CT examination to PB collection (1st/2nd/3rd)	DLP (mGy·cm)	WAZA-ARI (mSv)^b^ (1st/2nd/3rd)	Treatment^c^	Smoking status^d^	Past CT examination^e^	Past other X-ray examinations
1	Male	63	Lymphoma	Cervix, chest, abdomen, pelvis	391	380	8/16/7	2679.70/3695.85/3291.20	46.30/57.88/57.17	(+) Chemo	(−)	(+)	Chest, UGI, PET
2	Female	67	Lymphoma	Cervix, chest, abdomen, pelvis	331	350	9/19/7	1682.30/4354.12/4222.08	30.82/75.22/68.25	(−)^f^	(+)	(+)	Chest, UGI, PET
3	Male	64	Abnormal chest shadow	Cervix, chest, abdomen, pelvis	364	364	7/7/7	5856.00^a^/4119.80/3321.70	54.40/69.47/60.16	(−)	(+)	(+)	Chest, UGI
4	Male	51	Lymphoma	Cervix, chest, abdomen, pelvis	341	352	6/9/7	3319.80/3046.10/2635.20	61.83/50.67/46.72	(−)	(+)	(+)	Chest, UGI
5	Male	73	Lymphoma	Chest, abdomen, pelvis	490	364	7/14/14	2448.40/1990.50/1704.20	46.22/37.25/32.46	(+) Chemo	(−)	(+)	Chest, UGI, PET
6	Female	27	Lymphoma	Cervix, chest, abdomen, pelvis	153	358	6/15/14	1248.00/2059.93/1656.50	27.06/35.95/32.73	(+) Chemo	(−)	(+)	Chest, PET
7	Male	77	CML	Chest, abdomen, pelvis	445	280	11/14/31	3368.50/3064.30/2405.70	62.61/56.59/46.98	(+) TKI	(+)	(+)	Chest, UGI
8	Female	63	Lymphoma	Cervix, chest, abdomen, pelvis	742	700	14/7/7	3265.60/3113.00/2757.60	63.54/64.21/56.96	(+) Chemo, RT	(−)	(+)	Chest, UGI

^a^CTDI was defined using 16 cm phantom. All other CTDI were defined using 32 cm phantom.

^b^ Each effective radiation dose of the CT scan was calculated by the computational dosimetry system (WAZA-ARI).

^c^ Chemotherapy or radiotherapy had been performed at least 3 years before this study. Chemo = chemotherapy (mainly rituximab plus CHOP = cyclophosphamide, doxorubicin, vincristine and prednisolone); TKI = tyrosine kinase inhibitor; RT = radiotherapy

^d^ Patients 2, 4 and 7 had stopped smoking 1 year before this study. Patient 3 was still smoking at the beginning of the study.

^e^ All patients underwent CT scanning more than five times during the past 5 years.

^f^ Treatment with tumor resection without additional chemotherapy or radiotherapy.

### Staining

Chromosomes on each slide were stained with two methods. Centromere-fluorescence *in situ* hybridization (Centromere-FISH) was performed with the Poseidon probe (KRATECH, Amsterdam, The Netherlands) according to the manufacturer’s protocol with slight modifications as described in our previous study [8]. Chromosome painting was performed with the Customized XCP-Mix probe for chromosomes 1, 2 and 4 (Mix-#1R-#2G-#4RG; MetaSystems, Altlussheim, Germany) according to the manufacturer’s protocol.

### Image capturing and scoring

FISH images were captured in the AutoCapt mode using two sets of AXIO Imager Z2 microscopes (Carl Zeiss AG, Oberkochen, Germany) equipped with CCD cameras and Metafer 4 software (MetaSystems GmbH). Chromosome analysis was performed according to the International Atomic Energy Agency manual [11, 12] by three trained and experienced observers who were blinded to irradiated dose.

The Dics and Trs seen in FISH images were confirmed using fluorescence imaging software (Isis FISH Imaging System, ver. 5.4, MetaSystems GmbH). In total, more than 2000 metaphase chromosomes were scored in the Centromere-FISH slide [8]. Chromosomes were classified as dicentric or multicentrometric (chromosomes with three or more centromeres). Metaphase chromosomes with <45 centromeres were omitted from analysis. For the Trs analysis, >5000 metaphase chromosomes were scored in each sample [9]. Based on a previous report [13], we included apparently one-way Trs in the two-way Trs counts. In the case of complex chromosomal abnormalities, the numbers of Trs were determined based on the number of color junctions [14]. Other chromosome- or chromatid-type aberrations were also recorded such as rings, acentric fragments, breaks and gaps. For scoring, the formula used to calculate the frequency of Trs formation across the whole genome (F_G_) was based on the formula using three colors (chromosome 1: red, chromosome 2: green, chromosome 4: yellow) for the detection of translocations as follows [12]:

F_G_= F_P_(1+2+4) / 2.05 [f_1_(1-f_1_)+f_2_(1-f_2_)+f_4_(1-f_4_) - (f_1_f_2_+f_1_f_4_+f_2_f_4_)]

F_G_: the whole genome aberration frequency,

F_p_: the translocation frequency detected by FISH,

f_p_: the fraction of the genome hybridized, taking into account the gender of the subjects (female: f_p_ = 0.2234, male: f_p_ = 0.2271).

The proportion of the genome occupied by chromosomes 1, 2 and 4 is about 23%. Therefore, F_G_ is determined by the following formula:

F_G_ = F_P_ × 2.567 (Female)

F_G_ = F_P_ × 2.533 (Male)

To unify the cell numbers for the analysis, we determined F_G_ per 2000 cell equivalents, which were obtained according to the above formulas for females or males. For FISH slides, we switched to each captured filter image and carefully checked for the presence or absence of a Trs signal.

### Calculation of the effective CT scan radiation dose

A Toshiba Aquilion model 64 CT scanner was used in this study, with a tube voltage of 120 kV. The effective radiation dose was calculated by inputting data regarding age, sex and the initiation and end position of the CT scan into the computational dosimetry system (WAZA-ARI: http://waza-ari.nirs.go.jp/waza_ari/) [15–17]. Dose-length products (DLP) were extracted from parameters of the CT scan.

### Statistical analyses

The differences in the Dics and Trs frequency between before and after a CT examination were assessed using the Student’s paired t-test. A trend in the Dics and Trs frequency due to an ~1-year interval was also assessed. A *P*-value of <0.05 was considered significant. Multiple comparisons by Bonferroni’s method were performed to evaluate accumulation of chromosomal abnormalities by continuous CT examination. Specifically, the ‘adjusted significance level (α′)’ was obtained by the Bonferroni method, and the probability value of the Student’s paired t-test result of each comparison pair was judged by α′ (0.017). Analyses were performed using IBM SPSS Statistics 25 (IBM Corp, Armonk, NY, USA).

## RESULTS

### Patient background data

Patient background data are shown in Table 1. Eight patients were assessed, because not many patients remained in remission after chemotherapy and/or radiotherapy, and were followed by CT examination. The eight patients in this study were different from those in a previous study [8, 9]; five patients with malignant lymphoma (ML) were in remission and had not received treatment for more than 3 years after the end of treatment, one patient with ML received tumor resection treatment only, one patient had been followed due to an abnormal lung shadow without treatment, and another one patient with chronic myelogenous leukemia (CML) was receiving treatment with a tyrosine kinase inhibitor (TKI) and showed a major molecular response. This patient underwent consecutive CT examinations for the follow-up after resection of colorectal cancer. Four patients were smokers. CT examination was performed once a year except for patient 8 whose CT examination interval was about 2 years. All patients did not take upper gastrointestinal tract (UGI) examination or positron emission tomography (PET) examination during this research period. The effective radiation dose per CT examination estimated from the computational dosimetry system (WAZA—ARI) was 27.1–75.2 mSv, and the average dose per patient was 51.6 mSv (Tables 1 and 2).

**Table 2:** Results of dicentric chromosome and translocation analyses

Patient No.	Blood sampling^a^	DLP (mGy·cm)	Effective dose (mSv^c^	Analysis of dicentrics (Dics)				Analysis of translocations (Trs)				
				Cell counts	Dics	Frequency^d^	Increment	Cell counts	Cell equivalent^e^	Trs	Frequency^d^	Increment
1	1B	2679.70	46.30	2009	7	0.348	0.1	5120	2021	157	7.767	0.799
	1A			2008	9	0.448		5175	2043	175	8.566	
	2B	3695.85	57.88	2005	6	0.299	−0.050	5112	2018	122	6.045	2.382
	2A			2010	5	0.249		5110	2017	170	8.427	
	3B	3291.20	57.17	2009	7	0.348	0.101	5149	2033	166	8.166	0.188
	3A			2003	9	0.449		5124	2023	169	8.354	
2	1B	1682.30	30.82	2015	11	0.546	0.201	5211	2030	35	1.724	1.052
	1A			2007	15	0.747		5179	2018	56	2.776	
	2B	4354.12	75.22	2012	9	0.447	0.15	5139	2002	39	1.948	1.504
	2A			2009	12	0.597		5131	1999	69	3.452	
	3B	4222.08	68.25	2008	10	0.498	0.101	5132	1999	62	3.101	−0.199
	3A			2005	12	0.599		5130	1998	58	2.902	
3	1B	5856.00^b^	54.40	2011	9	0.448	0.2	5182	2046	155	7.577	2.199
	1A			2005	13	0.648		5234	2066	202	9.776	
	2B	4119.80	69.47	2006	8	0.399	0.149	5215	2059	198	9.617	−1.174
	2A			2008	11	0.548		5130	2025	171	8.443	
	3B	3321.70	60.16	2007	12	0.598	0.148	5105	2015	169	8.385	1.8
	3A			2012	15	0.746		5521	2180	222	10.185	
4	1B	3319.80	61.83	2007	8	0.399	0.045	5121	2022	40	1.979	1.293
	1A			2025	9	0.444		5264	2078	68	3.272	
	2B	3046.10	50.67	2003	6	0.3	0.1	5272	2081	59	2.835	0.435
	2A			2001	8	0.4		5654	2232	73	3.27	
	3B	2635.20	46.72	2000	7	0.35	0.099	5407	2135	61	2.858	0.207
	3A			2006	9	0.449		5289	2088	64	3.065	
5	1B	2448.40	46.22	2008	16	0.797	0.098	5520	2179	101	4.635	−0.368
	1A			2012	18	0.895		5402	2133	91	4.267	
	2B	1990.50	37.25	2005	16	0.798	0.15	5204	2054	99	4.819	0.656
	2A			2004	19	0.948		5228	2064	113	5.475	
	3B	1704.20	32.46	2002	20	0.999	0.151	5141	2030	84	4.139	−1.375
	3A			2000	23	1.15		5132	2026	56	2.764	
6	1B	1248.00	27.06	2001	5	0.25	0.149	5109	1990	95	4.773	−0.632
	1A			2005	8	0.399		5145	2004	83	4.141	
	2B	2059.93	35.95	2003	5	0.25	0.246	5273	2054	95	4.625	0.938
	2A			2015	10	0.496		5168	2013	112	5.563	
	3B	1656.50	32.73	2002	4	0.2	−0.050	5210	2030	48	2.365	1.178
	3A			2001	3	0.15		5216	2032	72	3.543	
7	1B	3368.50	62.61	2004	6	0.299	0.1	5192	2050	89	4.342	3.185
	1A			2003	8	0.399		5115	2019	152	7.527	
	2B	3064.30	56.59	2000	3	0.15	0.049	5205	2055	165	8.03	0.361
	2A			2006	4	0.199		5162	2038	171	8.391	
	3B	2405.70	46.98	2004	2	0.1	0.095	5397	2131	173	8.119	−1.037
	3A			2048	4	0.195		5222	2062	146	7.082	
8	1B	3265.60	63.54	2000	17	0.85	0.15	5112	1991	681	34.197	0.735
	1A			2000	20	1		5144	2004	700	34.932	
	2B	3113.00	64.21	2001	14	0.7	0.1	5232	2038	589	28.898	3.414
	2A			2002	16	0.8		5140	2002	647	32.312	
	3B	2757.60	56.96	2005	12	0.599	0.15	5137	2001	730	36.479	5.925
	3A			2003	15	0.749		5194	2023	858	42.404	

^a^ Blood sampling either before (B) or after (A) the first (1), second (2) third (3) CT examination.

^b^ CTDI was defined using 16 cm phantom. All other CTDI were defined using 32 cm phantom.

^c^ Estimated effective radiation dose (whole body exposure dose) according to ICRP 103 using WAZA-ARI.

^d^ Frequency in 100 cells.

^e^ Cell counts were converted to the equivalent number of cells as described in the Materials and Methods section.

### Frequency of Dics formation and effective radiation dose per CT examination

**Fig. 1:**
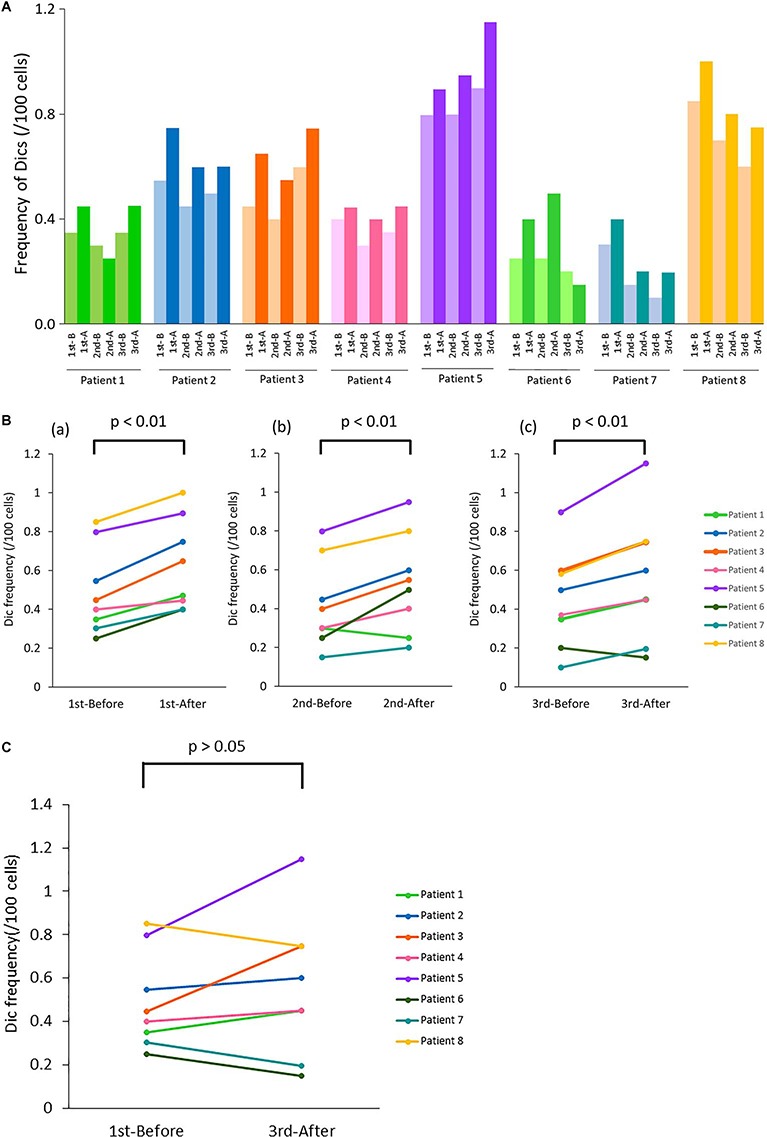
Frequency of Dics formation and changes in frequency with three consecutive CT examinations. (**A**) Analysis of Dics formation before and after each of three CT examinations. The light colored bar indicates the frequency before CT and the dark colored bar indicates the frequency after CT. 1st-B, 2nd-B and 3rd-B: before 1st CT, 2nd CT and 3rd CT, respectively. 1st-A, 2nd-A and 3rd-A: after 1st CT, 2nd CT and 3rd CT, respectively. (**B**) Comparison of the frequency of Dics formation before and after each CT examination. The frequency of Dics formation increased significantly after each CT examination: (a) 1st CT examination, *P* = 0.0003; (b) 2nd CT examination, *P* = 0.008; (c) 3rd CT examination, *P* = 0.007. (**C**) Comparison of the frequency of Dics formation before the 1st CT and after the 3rd CT. No significant difference was found (*P*> 0.05).

Actual analyzed cell number and the distribution of Dics number are shown in Supplementary Table 1 (see online Supplementary material). The frequency of Dics formation per 100 cells and effective dose per each CT examination are shown in Table 2. A total of 2000 were analyzed before and after each CT examination, the number of Dics formation was counted and the increase/decrease in Dics was calculated. Changes in the number of Dics formation converted to the equivalent per 100 cells are shown in Fig. 1A. As a general feature, the frequency of Dics formation increased significantly after the CT examination (Fig. 1B). Furthermore, no accumulation of Dics formation was detected after three consecutive CT examinations (Fig. 1C).

### Frequency of Trs formation and effective radiation dose per CT examination

**Fig. 2:**
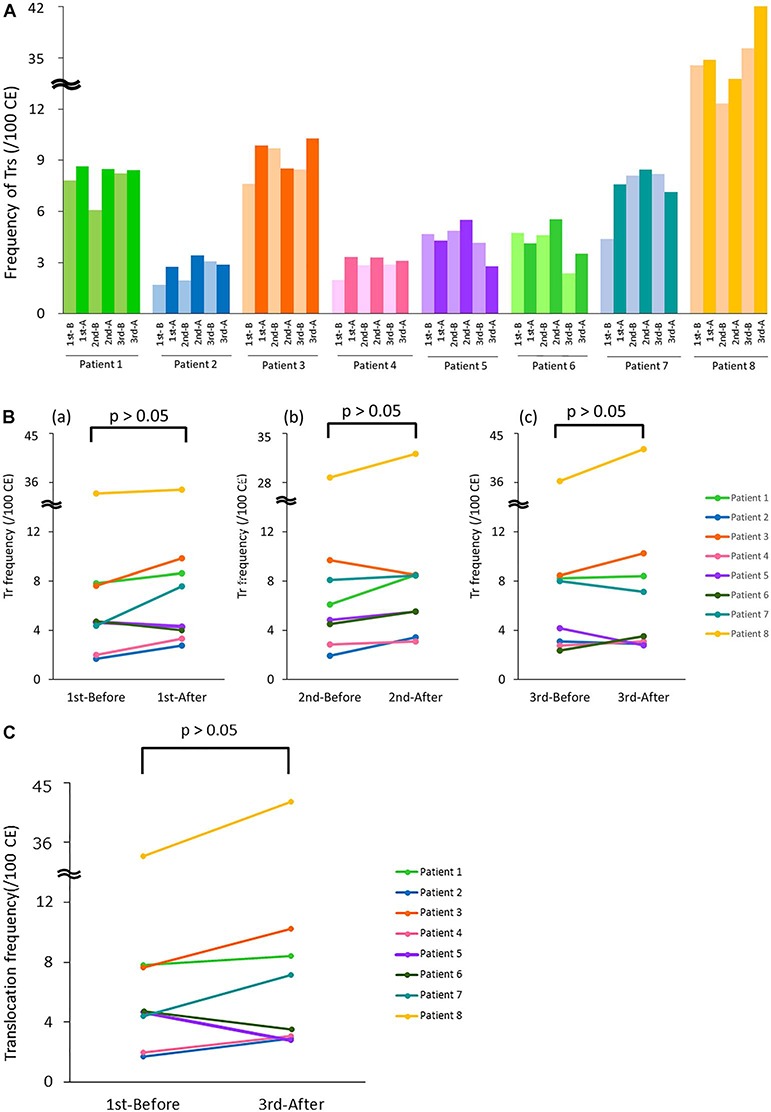
Frequency of Trs formation and changes in frequency with three consecutive CT examinations. (**A**) Analysis of Trs formation before and after each of three CT examinations. The light colored bar indicates the frequency of Trs formation before CT and the dark colored bar indicates the frequencies after CT. 1st-B, 2nd-B and 3rd-B: before 1st CT, 2nd CT and 3rd CT, respectively. 1st-A, 2nd-A and 3rd-A: after 1st CT, 2nd CT and 3rd CT, respectively. CE = cell number converted to the equivalent of 100 cells. (**B**) Comparison of the frequency of Trs formation before and after each CT examination. No significant difference was found in the frequency of Trs formation in each CT examination. (**C**) Comparison of the frequency of Trs formation before the 1st CT and after the 3rd CT. No significant difference was found (*P*> 0.05).

Actual analyzed cell number and the distribution of Trs number are shown in Supplementary Table 1 (see online Supplementary material). The frequency of Trs formation and effective dose per CT examination are shown in Table 2. To calculate the frequency of Trs formation in all chromosomes from those of chromosomes 1, 2 and 4, 5000 cells were analyzed, which corresponds to 2000 cells as in Dics analysis because the DNA content of chromosomes 1, 2 and 4 accounts for 23% of the DNA content in all chromosomes [12]. The frequency of Trs formation on all chromosomes, expressed as the frequency per 100 cells, and the increase/decrease in the frequency of Trs formation before and after each CT examination were determined. Changes in the number of Trs formation converted into the equivalent per 100 cells are shown in Fig. 2A. Unlike Dics analysis, we observed no significant increase in the frequency of Trs formation before and after the CT examination (Fig. 2B), and we found no accumulations of Trs formation after three consecutive CT examinations (Fig. 2C). The frequency of Trs formation was higher than that of Dics formation regardless of CT examination. We suspect that a cause of the high frequency of Trs formation in patient 8 regardless of CT examination (Fig. 2A) was the influence of the combination of chemotherapy and radiotherapy for lymphoma.

We also analyzed the relationship between the increment of chromosomal aberrations frequency and smoking status (Supplementary Table 2, see online Supplementary material) with reference to the research by Zhang *et al*. [18]. Although the number of samples was small, we could not find a significant relationship between them.

Therefore, a noteworthy point in this study is that a cumulative increase in the frequency of Dics and Trs formation after three consecutive CT examinations was not observed in the eight patients studied; the chromosomes in these patients may have been affected by aging, treatment for their disease and smoking.

## DISCUSSION

The important point of this study is that we analyzed whether Dics and Trs formation increase cumulatively with multiple CT examinations in which the radiation exposure dose per CT examination is <100 mSv. Although the number of analyzed cases was small, the analyzed 2000 genomes per sample for both Dics and Tr analysis was twice the number of analyses in the standard method.

In our previous study, we indirectly found that chromosomes are damaged by radiation exposure from a single CT examination, as shown by an increase in the frequency of Dics formation after CT examination [8]. However, we did not detect a significant increase in Trs formation [9], which are thought to be produced in about an equal ratio as Dics following ionizing radiation exposure [19, 20]. Because cells with Trs are mitotically stable, an increase in Trs induced by a CT examination could be hidden in the frequency of Trs formation due to various confounding factors. On the other hand, the frequency of Trs formation was higher than that of Dics formation both before and after CT examination [9]. The features of these chromosome aberrations were observed similarly in this study.

Cells with Dics are unable to survive for a long time because those cells are mitotically unstable and unable to undergo repeated cell division. The half-lives of cells with Dics were reported as 1–1.5 years in analyses using clinical samples exposed to ionizing radiation [21, 22]. However, a Dics analysis in a high background radiation area (HBRA) in China showed that an increase in Dics was correlated with the cumulative radiation exposure dose of residents [23, 24]. On the other hand, a study of a HBRA in Iran did not show a positive correlation as seen in the study in China [25]. The ionizing radiation exposure in a HBRA is a sustained, low-dose exposure, which is different from the repeated acute radiation exposure by CT examination that we examined in this study. The frequency of Dics formation increased transiently following a CT examination, and then decreased, and a cumulative increase in the frequency of Dics formation by three consecutive CT examinations was not observed. Previous studies revealed DNA DSBs using the Dics analysis or γ-H2AX foci staining after CT examination [26, 27]. The former showed that the mean number of Dics was correlated with the absorbed dose at CT examination under 9 years of age and the later showed that both the number of Dics and γ-H2AX foci were correlated with the absorbed dose when a fresh whole blood drawn from a healthy donor was exposed to CT scan. Although we did not find a correlation between the effective radiation dose and the frequency of Dics formation, our study, in which analyses of Dics and Trs at the same time of CT examination and further analyses of those changes after three consecutive CT examinations were performed, could be the first report.

Actually, the number of patients analyzed in this study may not be enough for this research. Furthermore, the patients analyzed have histories of chemotherapy and/or radiotherapy in the past, therefore, there is concern about genomic instability induced by treatment. However, what we emphasize in particular is that the number of Dics and Trs formed did not increase after three consecutive CT examinations even in patients suspected of having genomic instability. If the number of Dics and Trs formed increased in 8 patients analyzed, we should have further increased the number of patients or analyzed them in healthy individuals to find whether there was a truly significant difference.

Furthermore, small number of chromosomal aberrations induced by CT may be overlaid on the pre-existing aberration frequencies, which changes in a time-dependent manner after their formation, either by diminishing (Dics) or surviving (Trs) during lymphocyte proliferation, therefore, which depend on types of aberrations. Therefore, the present observation does not necessarily imply nil accumulation of DNA damage in at least stationary organs in the field of a CT scan.

Lymphocytes used for chromosome analysis are thought to be redistributed throughout the whole body from the exposed site because more than 1 day had passed after the CT examination. The distribution of lymphocytes at the time of CT examination could differ according to organ. The effective radiation dose of a CT scan is the dose specifically weighted for organs and tissues, and does not necessarily represent the dose absorbed by blood lymphocytes in those areas. Therefore, the effective radiation dose per CT examination did not directly correlate with the increasing number of both Dics and Trs appearing in PB lymphocytes. We also presented DLP in each CT examination to compare it with the equivalent CT scan radiation dose. Basically the machine dose of the CT apparatus is defined as the volume CT dose index (CTDI_vol_), which is the absorbed dose measured using a water phantom, including the correction factor of beam pitch to perform the evaluation of the CT apparatus. This CTDI_vol_ multiplied by the captured length of the CT scan is the DLP, and the DLP multiplied by the weighting factor (mSv x mGy^−1^ x cm^−1^) [28] is the equivalent dose. In a total of 24 CT examinations in 8 patients, the DLPs and effective CT scan radiation doses by WAZA-ARI were strongly correlated (data not shown). Exceptionally, those two factors were not correlated in second and third CT examination in patient 1 (Table 2). We suspect that the difference in CTDL_vol_ due to different parameters at CT examination was a cause.

Shi *et al.* analyzed chromosomal aberrations, dicentric and ring chromosomes in patients with ischemic heart disease before and after a CT scan procedure [29]. Similar to our results, they observed a significant increase in those chromosomes after CT examination. Although they emphasized that the analyzed subjects were non-cancer patients, they did not describe the patients’ backgrounds. We suspect that those patients also have smoking history, medical radiation history and life-related disease, which are usually thought to be a cause of chromosomal instability. Furthermore, they showed an increase in the number of dicentric and ring chromosomes in lymphocytes from four healthy volunteers. However, those subjects did not receive a CT examination and instead PB in tubes was irradiated with a CT scan. Therefore, the situation of ionizing radiation exposure for lymphocytes cannot be considered identical between patients and healthy volunteers.

A possible adverse effect of multiple CT examinations is the occurrence of malignant neoplasms. The use of CT examination for lung cancer screening contributed an estimated 0.05% to the prevalence of lung cancer over 10 years [30]. However, this rate probably does not reflect an actual risk in clinical practice for adults. On the other hand, another study showed no significant increase in the prevalence of cancer after excluding children with cancer-predisposing factors [7], suggesting that ionizing radiation exposure during a CT examination may be a cause of cancer only in children with cancer-predisposing factors.

Dics and Trs are chromosome aberrations that are not detected unless the double strands of DNA are cleaved. Another form of DNA damage caused by radiation exposure is gene mutation. Gene mutations are a major cause of solid tumor and myelodysplastic syndrome [31–33]. Ideally, we should perform genome analysis using next-generation-sequencing before and after a CT examination to help determine whether CT examination would be a risk factor for the onset of cancer.

Finally, although a CT scan is a very useful tool in medical care, it is necessary to consider the risk of excessive ionizing radiation exposure. Furthermore, because the data may contain uncertainties concerning the estimation of dosimetry and the limited number of cases, further study is needed to generalize the conclusions in the present study.

## Supplementary Material

Supplementary_Table_1_20190117_rrz068

Supplementary_Table_2_rrz068

## Notes

Presentation at the American Association for Cancer Research Annual Meeting 2018 in Chicago.
